# Management of Pediatric Urolithiasis in an Italian Tertiary Referral Center: A Retrospective Analysis

**DOI:** 10.3390/medicina59122165

**Published:** 2023-12-14

**Authors:** Francesco Lasorsa, Claudia Caliolo, Antonia Silecchia, Nicola Laricchiuta, Michele Raguso, Pasquale Ditonno, Giuseppe Lucarelli

**Affiliations:** 1 Department of Precision and Regenerative Medicine and Ionian Area-Urology, Andrology and Kidney Transplantation Unit, University of Bari “Aldo Moro”, 70124 Bari, Italy; 2 Urologic Pediatric Surgery Unit, Pediatric Hospital “Giovanni XXIII”, 70124 Bari, Italy

**Keywords:** pediatric urolithiasis, kidney stones, URS, RIRS, PCNL

## Abstract

*Background and Objectives*: In recent years, the prevalence of pediatric urolithiasis has increased in North America and Western countries, though it is endemic in developing countries. The aim of this study is to describe the experience of a tertiary pediatric referral center in the surgical management of pediatric urolithiasis. *Materials and Methods*: We retrospectively reviewed the experience of patients ≤ 16 years old affected by urinary stones who underwent surgery. *Results*: From April 2021 to September 2023, 31 pediatric patients underwent surgical procedures for stone diseases at our department: 13 preschool-aged (1–5 years) and 18 school-aged (6–16 years) children. During this period, 12 URSs, 17 RIRSs, and 2 PCNLs were recorded. Five patients had residual fragments at first, so three of them underwent a second endourological lithotripsy (2 RIRSs and 1 URS). Complete clearance was finally achieved in 27 patients. The stone composition was evaluated in 25 cases. *Conclusions*: Numerous innovations in the surgical treatment of pediatric urolithiasis have resulted from the development of smaller devices and new technology. Our results show how, in experienced centers, retrograde and percutaneous lithotripsy are safe and effective procedures for use in pediatric populations.

## 1. Introduction

In recent years, the prevalence of pediatric urolithiasis has increased in North America and Western countries, though it is endemic in developing countries. During the 2005–2016 period, approximately 65/100,000 pediatric individuals were estimated to have developed urolithiasis, compared to 18/100,000 cases in 1999 [1]. The causes are unclear, although several reasons have been proposed, including obesity, dietary modifications that increase salt intake, a decrease in calcium and water intake, and an increase in fructose consumption and the use of antibiotics. All ages and both genders may be equally impacted, even if female patients have a higher prevalence [2]. Even with management, some studies predict a 50% chance of symptomatic stone recurrence within 3 years. This rate is higher than that of adult cases. In the absence of any intervention, up to 75% of cases recurred after three years [3,4]. Warming weather, antibiotic exposure, and supplement use are incriminated as putative risk factors for stone recurrence [5]. The distribution of composition is comparable to that of adult stones, with 70–80% being calcium oxalate, 10–15% being struvite, 10% being calcium phosphate, and less than 5% being uric acid [6]. Over the years, affected children are at risk of developing comorbidities such as decreased bone mineral density (BMD), chronic kidney disease (CKD), and cardiovascular disease. Multiple stone formers had an increased risk of CKD by nearly eight times [7]. Besides inherited metabolic errors and congenital malformations, metabolic disorders can account for 50% of kidney stones in children under the age of 10. Anatomical risk factors include ureteropelvic junction obstruction, giant ureter, ureteral cyst, and urethral valves. It is of utter importance to perform a metabolic evaluation for children with nephrolithiasis. X-ray diffraction, infrared spectroscopy, or polarization microscopy can diagnose stone composition. Furthermore, spot urine samples or 24 h urine collection are demanded for urinary data on volume, pH, electrolytes (sodium and calcium), creatinine, oxalate, citrate, uric acid, and cystine. Serum levels of calcium, phosphorus, bicarbonate, magnesium, and uric acid should be determined as well. The most common metabolic anomalies are hypercalciuria, hyperoxaluria, hypocitraturia, cystinuria, and hyperuricosuria [8]. Hypercalciuria rates were 33.8–42% amongst pediatric patients, with idiopathic hypercalciuria (normocalcemia with hypercalciuria) being the leading form [9]. Primary and secondary forms of hyperoxaluria are described. Three rare autosomal recessive disorders are responsible for the increased liver production of oxalate owing to defective enzymes: alanine glyoxalate aminotransferase (type I primary hyperoxaluria—PH1), glyoxalate reductase-hydroxypyruvate reductase (PH2), and 4-hydroxy-2-oxoglutarate aldolase (PH3) [10]. Increased intestinal oxalate absorption (enteric oxaluria) or excessive dietary intake might result in secondary hyperoxaluria. Celiac disease, cystic fibrosis, inflammatory bowel diseases, and short bowel syndrome augment intestinal oxalate bioavailability because of fat malabsorption [11]. Since citrate can inhibit stone formation through different mechanisms, hypocitraturia is a risk factor for nephrolithiasis. Hyperuricosuria can occur in children treated for leukemia or lymphoma or in those with inborn errors in purine metabolism, such as hypoxanthine-guanine phosphoribosyltransferase (Lesh-Nyhan syndrome), adenine phosphoribosyltransferase, xanthine dehydrogenase, or phosphoribosylpyrophosphate synthetase [12]. A urinary pH below 5.5 promotes uric acid crystallization. Cystinuria is characterized by the defective reabsorption of cystine, arginine, ornithine, and lysine at the level of the proximal renal tubule, and it is transmitted in an autosomal recessive manner [13]. Cystine precipitates at a low urinary pH. The surgical management of pediatric urolithiasis includes extracorporeal shockwave lithotripsy (SWL), ureteroscopy (URS), retrograde intrarenal surgery (RIRS), and percutaneous nephrolithotomy (PCNL). The number, size, location, and composition of stones, as well as urinary system anatomy, available equipment, and surgeon experience, are crucial in the decision-making process about treatment options. Complete stone clearance, preventing recurrences, preserving renal function, controlling urinary tract infections, minimizing invasiveness, reducing anesthesia and radiation exposure, minimizing the number of surgical drainage procedures, and correcting underlying metabolic disorders and anatomic abnormalities should all be considered. In recent years, endourology has emerged as a safe and effective method for treating stones in children thanks to better equipment and growing expertise with retrograde and percutaneous treatments for adult patients [14,15,16]. The aim of this study is to describe the experience of a tertiary pediatric referral center in the surgical management of pediatric urolithiasis.

## 2. Materials and Methods

We retrospectively reviewed the experience of patients ≤ 16 years old affected by urinary stones who underwent surgery at the Pediatric Urologic Unit in collaboration with the Urology, Andrology and Kidney Transplantation Unit at the University of Bari. This study was conducted according to the Declaration of Helsinki and national and international guidelines; it was approved by the authors’ institutional review board (SN18/2023–7 October 2023). Written informed consent to take part was given by all participants.

Before surgery, preoperative blood tests and urine cultures were performed. Non-contrast computed tomography (CT scan) or kidney–ureter–bladder (KUB) radiographs/ultrasounds were used to assess the stone localization and size. Holmium: YAG laser fibers were used without active dilation of the distal ureter. Preoperative antibiotic prophylaxis with cephazolin or ceftazidime was administered. Pre-stented patients received low-dose antibiotic prophylaxis with cefixime. Depending on the duration of the procedure, the severity of ureteral edema, and/or the presence of residual fragments, ureteral catheters and—in the case of PCNL—nephrostomy tubes were left in place following surgery, whereas bladder catheters were kept for 24–48 h. Postoperative complications were described according to the Clavien–Dindo classification.

## 3. Results

From April 2021 to September 2023, 31 pediatric patients underwent surgical procedures for stone diseases at our department: 13 preschool-aged (1–5 years) and 18 school-aged (6–16 years) children. The median age was 7 years (range 48–144 months). Ten children were diagnosed with at least one metabolic disorder during screening: four hypercalciuria, one hyperoxaluria, five hypocitraturia, and one hyperaminoaciduria (Table 1). Comorbidities included growth retardation (*n* = 2), Lesh–Nyhan syndrome (*n* = 1), West syndrome (*n* = 1), cerebral palsy (*n* = 1), Duchenne muscular dystrophy (*n* = 1), and hypothyroidism (*n* = 1). Apart from these cases, the remaining 24 patients had no evidence of concomitant diseases. Three patients were chronically administered drugs: vigabatrin, allopurinol, baclofen, nitrazepam, perindopril, and bisoprolol. Although it was associated with the risk of xanthine lithiasis in children, we did not suspect allopurinol-induced stones or other drug-induced stones. Symptom onset occurred with pain (*n* = 18), hematuria (*n* = 13), or fever (*n* = 4). In two cases, urolithiasis was accidentally diagnosed, with the patient being asymptomatic. The median stone size was 10 mm, and the stone area range was 38.5–110.3 mm^2^. A group of 14 patients were diagnosed with concomitant hydronephrosis, and 12 of them were pre-stented before elective lithotripsy. The severity of patients’ clinical manifestation (unresponsive pain or fever) was the main indication for urgent stenting. Most of them were admitted to our department after accessing the emergency department (ER). As mentioned above, lithotripsy was performed electively after obtaining the results of a preoperative urine culture. Tailored antibiotic therapy is recommended in cases of positive preoperative culture to reduce the risk of sepsis. This is another crucial element that we take into consideration when stenting patients. Strikingly, all preoperative urine cultures tested negative in our series. During this period, 12 URSs, 17 RIRSs, and 2 PCNLs were recorded (Table 2). Because of ureteral stricture, one procedure was not completed. A ureteral access sheath (UAS) was used in 11/17 RIRSs, whereas a basket was used in 20 procedures to remove fragments. At the end of the lithotripsy, 8 mono J and 22 double JJ ureteral catheters were placed. In cases of PCNL, the nephrostomy tube was removed after 3 days. After the first procedure, the median time of indwelling for the stent was 31 days (range 22.4–36.6). Five patients had residual fragments at first, so three of them underwent a second endourological lithotripsy (2 RIRSs and 1 URS). Complete clearance was finally achieved in 27 patients. The stone composition was evaluated in 25 cases: calcium oxalate (51.6%), calcium oxalate/phosphate (6.5%), calcium oxalate/struvite (6.5%), calcium phosphate/struvite (3.2%), struvite (3.2%), and uric acid (9.7%). Two patients developed grade I complications (fever and stent removal). On postoperative day two, the patient diagnosed with West syndrome experienced fever (up to 39.6 °C), respiratory distress (oxygen saturation of 87%), and high serum inflammation markers. He underwent URS for a 32 mm stone in the distal ureter in the absence of prior stenting. As a result, he was temporarily admitted to the ICU, where he received adequate oxygen and antibiotic therapy (Clavien IV).

## 4. Discussion

Different interventional management is recommended by the 2023 European Association of Urology (EAU) guidelines, mainly based on stone size and localization: SWL, URS, RIRS, PCNL, and open or minimally-invasive surgery (nephrolithotomy, pyelolithotomy, or ureterolithotomy) [17,18]. Notably, the role of open surgery has been limited owing to the introduction of laparoscopic and robot-assisted surgeries. They should be considered in case of upper tract anomalies (UPJ obstruction, calyceal diverticula, and megaureter) to be repaired simultaneously, complicated renal anatomy (retrorenal or ectopic colon), or previous failed lithotripsy. Pediatric SWL was first reported in 1986 [19,20,21], and electrohydraulic, electromagnetic, and piezoelectric generators are available as sources of shock waves. Many renal and ureteral stones can be managed with SWL, especially those < 1 cm in diameter. SWL is also indicated for non-lower calyx stones (1–2 cm in diameter and <750 Hounsfield unit value) and upper (<1.5 cm), middle, or lower ureter (<1.0 cm) stones. SWL offers more advantages in children than in adults. First, the shorter skin-to-stone distance results in less shock wave energy attenuation. Second, the amount of water in the tissues between the body surface and the kidney is higher, and the acoustic impedance is low, both of which are favorable for energy transmission in a shorter ureter [22]. Coagulation disorders, cardiopulmonary diseases, uncontrolled urinary tract infections (UTIs), active infectious diseases, skeletal malformations, lithotripsy passage obstruction, obesity, CKD, and renal hemangiomas contraindicate SWL in children. Due to stone fragments, ureteral obstruction is the most common complication. UTIs can be avoided with adequate preoperative antibiotic therapy, even if perioperative prophylaxis is controversial. On the contrary, excessive numbers of shockwaves are indicated to be the cause of subcapsular renal hematoma. Another aspect under debate is the need for stent placement before the beginning of SWL sessions. However, SWL was found to be less successful for hard (cystine and calcium oxalate) and caliceal stones, in particular, lower caliceal stones, despite being less invasive. Stone-free rates are also affected by the size, and large stones demand multiple SWL sessions under general anesthesia [23].

For kidney stones > 2 cm in diameter, complete or partial staghorn stones, and lower calyx stones > 1 cm, PCNL is advised for the first-line treatment, as it is for adults. PCNL is the most invasive surgical procedure and requires a working sheath (to be positioned after dilation of the entry tract) and a rigid or flexible nephroscope. In 1985, the first pediatric case demanded instruments of an adult standard PNCL: a 30 Fr working sheath, a 24 Fr nephroscope, and laser or lithotrite devices. Various types of energy for intracorporeal lithotripsy can be used during PCNL, such as ultrasonic and pneumatic systems, holmium, or thulium lasers. Although the stone-free rate following PCNL in children has been reported to be as high as 68–100%, post-procedural complications (including fever, sepsis, renal pelvic perforation, persistent urine leakage, bleeding necessitating transfusion, and bowel injury) can occur, particularly when using adult-sized instruments on preschool-aged children [24]. The supine PCNL carries many advantages over the classical prone position [25]. Over the years, different supine positions have been described that are suitable for both adult and pediatric individuals: the flank-free modified supine position (FFMSP), complete supine percutaneous nephrolithotomy (csPCNL), the Galdakao-modified Valdivia position, and Giusti’s position [26]. Supine positions are associated with adequate respiratory compliance and better drainage to reduce fluid overload, which are crucial concerns in children. In turn, they offer more limited puncture areas than prone procedures. In children undergoing PCNL, a sheath size > 20 Fr has been reported as an independent factor for complications and bleeding [27]. Despite miniaturized access sheaths being first designed for pediatric patients, their use has been extended to all age groups: miniPCNL (access sheath size 14–20 Fr), ultraminiPCNL (11–13 Fr), and microPCNL (4.85 Fr access needle). A recent study showed that no blood transfusion or renal function impairment occurred in 20 children who underwent miniPCNL [28]. Compared to RIRS, miniPCNL showed improved stone-free rates and increased hospitalization and fluoroscopy use [29]. The main drawback of miniaturized PCNL is stone burden: <2.5 cm for miniPCNL and <1.5 for microPCNL.

The first attempt to extract lower ureteric stones in children through URS dates back to 1988. Semi-rigid URS (4.5/6 Fr, 6/7.5 Fr, and 8/9.8 Fr) allows for navigation into the whole ureter and even into the pelvicalyceal system. The development of flexible ureteroscopy (FLURS) has facilitated access to lower calyceal stones because of its maneuverability and wide range of deflection [30]. In parallel, RIRS can be adopted for upper ureteric or renal stones < 2 cm. It is feasible in the case of congenital skeletal or kidney malformations and lower calyceal stones when SWL is not indicated. Severe ureteral strictures, a narrow ureter, and an acute infundibulopelvic angle (IPA < 30°) limit its application [31]. Staghorn calculus or mutliple stones can benefit from endoscopic combined intrarenal surgery (ECIRS) to reduce operative time and anesthesia. Additional studies are still needed to validate the outcomes of ECIRS in children. The complication rate for pediatric URS varies from 1.3% to 5.2%, whereas success rates range from 77% to 100%. Because of its efficacy and low morbidity, RIRS emerged as a first-line treatment, although complication rates are higher in children < 5 years of age [32]. Nevertheless, the number of studies evaluating the safety and efficacy of RIRS is limited. Unsal et al. reported a serious complication rate of 5.8% and an 88% success rate after a single session of the RIRS procedure in preschool-aged children [33]. Eventually, three patients (9.7%) experienced postoperative complications in our series. None of the pre-stented patients developed complications, likely related to better ureteral compliance with endoscopic procedures. We did not note any significant difference in stone clearance between pre-stented and non-pre-stented children. Complete clearance was achieved in 83.9% of patients after the first procedure and in 87.1% of patients after the second. In adult individuals, UAS reduces intrarenal pressures and operative time and improves stone-free rates. Indeed, it keeps a clear vision since URS can be repeatedly removed and reintroduced, thus allowing dust and fragments to flow out. UAS insertion can be facilitated by pre-stenting. Due to the possible danger of ureteral injury (ureteral lacerations, strictures, avulsion, or vesicoureteral reflux), the use of UAS in the pediatric population is controversial [34]. No hydronephrosis nor recurrent urinary infections were registered in our patients. Active dilation of the ureteral orifice via balloon dilators or coaxial dilators is known to be a risk factor for ureteral ischemia, perforation, ureterovesical junction perforation, ureterovesical reflux, or ureteral stricture. Active dilation was not carried out in any of our patients. One patient underwent double JJ stent placement after the first initial failed attempt at upper urinary tract access. In this field, stent placement after surgery is under debate. It reduces pain and infection due to residual fragments, but it requires readmission for its removal.

General dietary advice should be offered to patients: adequate hydration (1000 mL/1.73 m^2^/day), a low-sodium diet, or reduced oxalate intake (chocolate and peanuts). Specific dietary and medical intervention is necessary if there are underlying metabolic disorders that raise the risk of recurrences. Thiazides may also be beneficial for hypercalciuria. Potassium citrate is indicated in case of hypocitraturia or hypercalciuria, for instance. Treatment for uric acid stones involves potassium citrate to prevent stone formation by modifying urine pH and allopurinol to restore serum uric acid levels. Patients diagnosed with cystinuria may be administered tiopronine to decrease cystine urinary excretion [35].

Spontaneous expulsion of distal ureteral stones can occur, and its likelihood is associated with their number, size, location, structure, smooth muscle spasms, and ureteral edema. Expectant management and metabolic screening should be offered in case of small, asymptomatic, and non-obstructing stones. A blockade of α1-adrenoreceptors (mainly subtype D) inhibits hyperperistaltic uncoordinated frequency while preserving ureteral tonic propulsive contractions, producing lumen dilation and possible stone elimination [36]. As a result, evidence supports the use of α-blockers as a medical expulsive therapy (MET) in adults, but the literature relating to children is limited [37]. Different previous studies already proved the safety of these drugs for children’s voiding dysfunction. Compared with controls, a meta-analysis revealed that α-blockers increase the risk of large stone passage, especially in the lower ureter [38]. Tamsulosin resulted in a significantly higher stone expulsion rate (SER), a shorter expulsion time (SET), fewer pain episodes, and reduced analgesic need [39]. In turn, no significant difference was noted with doxazosin and ibuprofen [40]. However, in a prospective, randomized, placebo-controlled study, silodosin was more effective than tamsulosin as a MET for ureteric stones [41]. Additionally, tamsulosin has also been investigated as a preoperative therapy to reduce the rate of access failure during URS with or without UAS insertion, although more prospective and randomised studies are needed to confirm these preliminary findings [42]. For the acute management of fever and pain, only paracetamol and ibuprofen are available to children. Pain control versus adverse effects should be taken into consideration when administering pain relief, albeit ibuprofen appeared more effective [43].

When collecting a patient’s medical history, drug-induced calculi should be ruled out, even if they are rare in children. Two different mechanisms are described: drug-containing stones or metabolically drug-induced stones [44]. In the former scenario, urine supersaturation of the drug or its metabolites leads to its crystallization. The first category encompasses sulphonamides (low solubility in acidic urine), triamterene, felbamate, silica-containing antiacids, indinavir (poor solubility in alkaline urine), and ceftriaxone. A variety of pathophysiological mechanisms have been demonstrated for the second category of drugs. Furosemide causes hypercalciuria via a blockade of calcium tubular reabsorption. Carbonic anhydrase inhibitors acetazolamide and dorzolamide reduce bicarbonate reabsorption and alkalinize urine. Topiramate and zonisamide cause systemic metabolic alterations such as metabolic acidosis, elevated urinary pH, hypocitraturia, and hypercalciuria [45]. Allopurinol is administered to control hyperuricemia in gout and Lesch–Nyhan syndrome and prevent tumor lysis syndrome, as in acute lymphoblastic leukemia (before chemotherapy induction). By inhibiting xanthine oxidase, allopurinol increases xanthine urinary elimination, which can crystallize with or without oxypurinol (allopurinol metabolite) [46]. A ketogenic diet can raise the odds of urolithiasis through different events such as hypocitraturia, hypercalciuria, chronic acidosis, and dehydration, above all [47].

Blood urea nitrogen and serum creatinine are traditionally monitored to estimate the glomerular filtration rate (GFR) during acute kidney injury (AKI). However, their levels are influenced by different factors and slowly detect GFR changes. Considering several gene upregulations during AKI, a putative marker should be released early by the kidney and found in the blood or in the urine without further metabolism [48,49]. It has been largely described as the crucial role of complement system activation during kidney damage [50]. Urinary tract obstruction may lead to tubular atrophy and interstitial fibrosis through different mechanisms. It is well understood that recovery of renal function following ureteral obstruction relief is dependent on several parameters, including the location and duration of the obstruction, whether it is complete or partial, and concomitant infection. Thereafter, delayed relief decreases long-term renal function and increases the risk of arterial hypertension or its exacerbation. Integrating genomic, transcriptomic, proteomic, and metabolomic data can provide a deeper understanding of the molecular mechanisms of renal damage progression. Lucarelli et al. suggested that the urinary epidermal growth factor-1 (EGF)/monocyte chemotactic peptide-1 (MCP1) ratio could be a marker of progressive renal damage, for instance [51]. Low-molecular weight proteins (α1-microglobulin or β2-microglobulin) are widely recognized urinary markers for CKD [52]. Different studies pointed out that β2-microglobulin dramatically rises after SWL [53]. Therefore, it would be beneficial to identify and validate serum or urinary biomarkers to predict early renal damage, even in children with urolithiasis [54]. Overall, the early detection of tubular injury can facilitate and improve treatments.

In recent years, the introduction of high-throughput technologies has provided significant insights into the understanding of normal and pathological processes, as well as the identification of potential targets for the development of drugs to treat diseases such as cancer [55,56,57,58,59,60,61]. In particular, the analysis of low-molecular-weight metabolites (the metabolome) allows for the global assessment of a cellular state in normal and pathological conditions. This approach can be used to define the “metabolic fingerprint” of a tumor and identify novel biomarkers that may be potentially useful for both early diagnosis and monitoring the therapeutic response [57,58]. Moreover, studying the metabolome could provide clues to systemic or intrarenal differences that influence unrecognized steps in kidney stone pathogenesis. A metabolomics approach can be used to investigate the serum and urine of patients with kidney stones, with the aim of discovering abnormal metabolic pathways involved in urolithiasis and identifying novel markers for the diagnosis of kidney diseases associated with this condition [62]. A recent study evaluated the metabolite profiles of serum from children with urolithiasis and normal controls using ultra-performance liquid chromatography–mass spectrometry [63]. The metabolomic analysis of serum samples showed that steroid hormone biosynthesis was altered in children with urolithiasis. In particular, dihydrotestosterone, estradiol, and progesterone were significantly accumulated in children with kidney stones. On the contrary, bilirubin was significantly decreased. Bilirubin is an endogenous antioxidant that may have significant protective effects against the oxidative stress induced by crystals in renal tubular epithelial cells [64,65]. Recent studies have shown that bilirubin not only reduces renal injury by inhibiting oxidative stress and apoptosis but also promotes the proliferation of renal tubular epithelial cells [66,67]. These findings highlight the protective effect of bilirubin on renal tubular epithelial cells, and its deficiency in children with kidney stones may represent a promoting factor that increases crystal-associated damage in tubular epithelial cells. Interestingly, the all-transretinoic acid (ATRA) serum levels were reduced in children with kidney stones compared to normal controls. ATRA is the main active downstream product of the retinol metabolic pathway, and it is a kidney-protective factor [68]. In the kidney, tRA confers a protective effect by reducing the expression of inflammatory cytokines and complement system activation, protecting proximal renal tubular epithelial cells against ischemia-reperfusion injury and promoting the differentiation of renal tubular epithelial cells in response to renal tubular damage [50,69,70,71]. These findings provide a novel insight into the pathogenesis of urolithiasis in children and show how the metabolomic approach is a valid tool to identify novel therapeutic targets and develop preventive strategies in high-risk patients.

## 5. Conclusions

Numerous innovations in the surgical treatment of pediatric urolithiasis have resulted from the development of smaller devices and new technology [72]. The crucial concerns of surgical management in the pediatric population are reducing radiation exposure and adverse effects on the developing kidney and aiming for a single-step treatment. In experienced centers, retrograde and percutaneous lithotripsy are safe and effective. Identifying underlying conditions (anatomic variants or metabolic disorders) is crucial for an early diagnosis, tailored management, and reducing recurrences. Urologists, nephrologists, and general pratictioners should cooperate to improve patients’ quality of life, especially in this particular population of patients.

## Figures and Tables

**Table 1:** Demographic and preoperative date. US: ultrasounds; CT: computed tomography; UPJ: uretero-pelvic junction; GFR: glomerular filtration rate.

Data	Patients
Age, median months	84 (48–144)
Sex, *n* (%)	
Male	13 (41.9)
Female	18 (58.1)
Comorbidities, *n* (%)	7 (22.6)
Growth retardation	2 (6.4)
Lesh–Nyhan	1 (3.2)
West	1 (3.2)
Duchenne	1 (3.2)
Cerebral palsy	1 (3.2)
Hypothyroidism	1 (3.2)
Metabolic abnormalities, *n* (%)	
Hypercalciuria	4 (12.9)
Hyperoxaluria	1 (3.2)
Hypocitraturia	5 (16.1)
Hyperuricosuria	2 (6.4)
Hyperaminoaciduria	1 (3.2)
Cystinuria	0
Clinical presentation, *n* (%)	
Pain	18 (58.1)
Hematuria	13 (41.9)
Fever	4 (12.9)
Asymptomatic	2 (6.5)
Side, *n* (%)	
Left	15 (48.4)
Right	16 (51.6)
Imaging, *n* (%)	
US	4 (12.9)
X-ray	1 (3.2)
CT	26 (83.9)
Location, *n* (%)	
Pelvis	7 (22.6)
UPJ	3 (9.7)
Upper ureter	3 (9.7)
Lower ureter	9 (29.0)
Calyx	7 (22.6)
Staghorn	2 (6.5)
Size, mm, median	10 (7.0–12.4)
Area, mm^2^, median	62 (38.5–110.3)
Hounsfield, UI, median	550.5 (339.1–1134.6)
Preoperative stenting, *n* (%)	12 (38.7)
Preoperative stent indwelling time, median days	39 (19.6—60.0)
Preoperative creatinine, median mg/dL	0.41 (0.3—0.5)
Preoperative GFR, median mL/min	179 (149.8—188.0)

**Table 2:** Operative and postoperative outcomes.

Data	Patients
Type of surgery, *n* (%)	
URS	12 (38.7)
RIRS	17 (54.8)
PCNL	2 (6.5)
Intraoperative complications, *n* (%)	1 (3.2)
Residual fragments, *n* (%)	5 (19.4)
Reintervention, *n* (%)	3 (14.3)
Stone free, *n* (%)	
Yes	27 (87.1)
No	4 (12.9)
Stent removal, days, median	31 (22.4–36.6)
Stone composition	
Ca oxalate	16 (51.6)
Ca oxalate/Ca phosphate	2 (6.5)
Ca oxalate/struvite	2 (6.5)
Ca phosphate/struvite	1 (3.2)
Struvite	1 (3.2)
Uric acid	3 (9.7)
Unknown	6 (19.4)
Complications, *n* (%)	
Clavien I	2 (6.5)
Clavien II	0
Clavien III	0
Clavien IV	1 (3.2)
Clavien V	0

## Data Availability

No new data were created.
